# Seroprevalence, Detection of DNA in Blood and Milk, and Genotyping of *Toxoplasma gondii* in a Goat Population in Italy

**DOI:** 10.1155/2013/905326

**Published:** 2013-09-04

**Authors:** Francesca Mancianti, Simona Nardoni, Carlo D'Ascenzi, Francesca Pedonese, Linda Mugnaini, Filomena Franco, Roberto Papini

**Affiliations:** Dipartimento di Scienze Veterinarie, Università di Pisa, Viale delle Piagge 2, 56100 Pisa, Italy

## Abstract

*Toxoplasma gondii* is the causative agent of a major zoonosis with cosmopolitan distribution and is known to be transmitted mainly by the ingestion of undercooked or raw animal products. Drinking unpasteurized goat's milk is a risk factor associated with human toxoplasmosis. However, very little is known about the excretion of DNA in goat milk. Aim of the present study was to determine the seroprevalence of *T. gondii* infection using a modified agglutination test (MAT), to detect *T. gondii* DNA by nested-PCR (n-PCR) in samples of blood and milk from seropositive goats, and to genotype DNA isolates using 11 molecular markers in 127 adult lactating goats from 6 farms in Italy. Positive MAT results were found in 60.6% of goats while 13% of blood and milk samples from seropositive goats were positive to n-PCR. A kappa coefficient of 1 indicated a perfect agreement between blood and milk n-PCR. Genetic characterization of isolates revealed the occurrence of genotype III (*n* = 7), genotype I (*n* = 1), and atypical genotypes with hints for genotype I (*n* = 2). Our results suggest that the risk of excretion of *Toxoplasma* tachyzoites might frequently occur in milk of seropositive goats testing positive to n-PCR on blood.

## 1. Introduction

Toxoplasmosis is a zoonotic infection caused by *Toxoplasma gondii*, an opportunistic protozoan belonging to the phylum Apicomplexa. The population structure of *T. gondii* is complex and shows distinct geographic patterns [1]. Approximately 90% of the *T. gondii* isolates from Europe and North America can be classified into three distinct clonal lineages (types I, II, and III strains) that differ remarkably in pathogenicity [2, 3]. In addition, a fourth clonal lineage has been revealed in Nord America [4] and a higher genetic variability has been detected based on newer markers for genetic characterization [5]. The acute pathogenicity of *T. gondii* strains has been characterized in murine infections. The high pathogenicity of type I lineage is well represented by the commonly used laboratory strain RH, since the primary inoculation of a single infectious organism into a mouse is the cause of death in 8–12 days [2]. Instead, lineages II and III are considered low pathogenic in the mouse model [3]. *T. gondii* infections are widely prevalent in humans and animals worldwide [6]. Up to one-third of the human population in the world is chronically infected [6], and toxoplasmosis has been targeted one among the five parasitic diseases as priorities for public health action [7]. Human infections are primarily asymptomatic, but lymphadenopathy or ocular toxoplasmosis may occur in some patients. *T. gondii* infection in pregnant women may lead to abortion, stillbirth, or other serious consequences in newborns [8, 9]. In immune-compromised patients, toxoplasmosis can be fatal if not treated, and the reactivation of a latent infection can cause life-threatening encephalitis [8]. The parasite may be transmitted from a definitive to an intermediate host and *vice versa*, as well as between different definitive hosts or between different intermediate hosts [9]. Among food animals, sheep and goats are well-known sources of human infection [6]. Ingestion of undercooked goat meat and unpasteurized goat milk is considered source of human infections [6, 10]. Moreover, tachyzoites of *T. gondii *have been found in the milk of several species, including sheep, goats, and cows [9, 11]. Excretion of tachyzoites in the milk of naturally infected goats was also reported [12], and infections in family clusters by consumption of raw goat milk have been documented [13, 14]. Among food animals, goats appear to be more susceptible to clinical toxoplasmosis, and adult goats were reported to have died of acute toxoplasmosis [9]. The disease is a common cause of abortion and neonatal mortality in goats [6].

Serological screening in goats in Europe showed prevalence values of 15.2% [15] and 25.1% [16], further supporting the likelihood of infection by milk ingestion from this species. The epidemiology of toxoplasmosis in goats has not been exhaustively studied in Italy. The lack of studies involving the caprine milk as a potential source of toxoplasmosis transmission in this country highlights the importance of studying this species milk. The present study aimed to determine (a) the seroprevalence of *T. gondii* infection, (b) the occurrence of *T. gondii* DNA in blood and milk samples as an indication of the risk of excretion of the parasite in milk, and (c) the genotyping of *T. gondii* in a population of goats in Italy.

## 2. Materials and Methods

### 2.1. Animal Population Examined

Between April and May 2012, 127 adult (age ≥ 1 yr) lactating Chamoisee (Shamwahzay) brown goats were included in the study. The animals were from 6 (I to VI) randomly selected goat farms in the province of Massa-Carrara (44°2′0′′N 10°8′0′′E) in Tuscany, Central Italy. They included 59 subjects in farm I, 20 in farm II, 15 in farm III, 13 in farms IV and V, and 7 in farm VI. The animals examined represented the total number of goats within each herd, were raised for milk production, and were kept in extensive breeding with grazing all over the year. 

### 2.2. Blood, Serum, and Milk Sampling

Approximately 10 mL of blood was collected from the jugular vein of each animal into Vacutainer tubes with and without EDTA. Samples with EDTA were immediately stored and kept under refrigeration until arrival at the laboratory and further processing for the blood nested-PCR (n-PCR) procedure within 24–48 hours. Blood samples without anticoagulant were allowed to clot at room temperature for approximately 15 to 30 minutes. After samples were completely clotted, the sera were separated and kept under refrigeration until arrival at the laboratory, where they were further processed for serological examination within 24–48 hours. Concomitantly with the blood and serum collection, milk was also individually collected from all goats by manual milking of all teats using gloves during manipulation. Before collection, the teats were accurately cleansed with iodine alcohol. From the total quantity of milk each goat produced by manual milking, an aliquot of 50 mL was individually collected in a sterile centrifuge tube. Milk aliquots were then stored and kept under refrigeration until arrival at the laboratory, where they were stored at −80°C.

### 2.3. Serological Examination

Serum samples were examined for anti-*T. gondii *IgG antibodies via the modified agglutination test (MAT) using a commercial kit (Toxoscreen DA, Biomérieux, Lyon, France) in accordance with the manufacturer's instructions, and starting at a 1/20 dilution. Sera showing an agglutination titer of 1/20 or higher were considered positive and were end-titrated using 2-fold dilutions. The choice of this threshold value was a slight modification of a method previously described [16], to satisfy laboratory requirements.

### 2.4. DNA Extraction

After results of serological tests were known, blood and milk samples from seropositive goats were processed for DNA extraction and subsequent amplification by n-PCR while samples from seronegative goats were discarded. Milk contains minor amounts of nucleated cell in comparison to whole blood, so, after thawing at room temperature and prior to extraction of DNA, a concentration of milk samples was carried out by centrifuge at 2200 g for 5′ as reported by other authors [17]. To avoid interference by casein, 1 mL of pellet was treated with 200 *μ*L TE (1 mM EDTA, 10 mM Tris-HCl (pH = 7.6)) and 300 *μ*L 0.5 M EDTA (pH = 8), resuspended and centrifuged at 3000 g for 10′, as previously reported [18]. Milk samples were then diluted in 200 *μ*L of PBS. DNA was extracted from samples of both blood and milk using the QIAamp DNA minikit (Qiagen, Milan, Italy) in accordance with the manufacturer's instructions. After extraction, DNA was stored at −20°C until use.

### 2.5. Nested PCR

The n-PCR assay used to screen blood and milk samples for *T. gondii* DNA has previously been described [19]. Nested-PCR generally has a risk of cross-contamination from genomic DNA. In order to detect such cross-contamination in PCR and DNA extraction reagents, a negative control was added every 10 samples processed. Negative controls were prepared when DNA from samples analyzed was added to the master mix. They were prepared as separate samples where aliquots of sterile water were added to master mix and contained every reagent with the exception of the template DNA. Negative control reactions were setup and amplified identically to the n-PCR and performed alongside each assay.

### 2.6. Genetic Characterization of *T. gondii* Isolates from Goats

Direct genotypic characterization of *T. gondii* strains was performed by PCR amplification of 11 genetic markers (SAG1, 3-SAG2, alt. SAG2, SAG3, BTUB, GRA6, C22-8, C29-2, L358, PK1, and Apico) and thereafter was analyzed by restriction fragment-length polymorphism, as described by other authors [20].

### 2.7. Statistical Analysis

Prevalence values were calculated as number of positive animals/number of examined animals × 100 with the corresponding 95% confidence intervals (95% CI). Kappa coefficient was used as a measure of agreement between blood n-PCR and milk n-PCR. The following ranges were considered for interpretation of the kappa coefficient: poor agreement = less than 0.00, slight agreement = 0.00–0.20, fair agreement = 0.21–040, moderate agreement = 0.41–0.60, substantial agreement = 0.61–0.80, and almost perfect agreement = 0.80–1.00.

## 3. Results 

### 3.1. Serological Prevalence

Seropositive goats were found in all 6 farms. Overall, 77 out of 127 (60.6% [95% CI: 52.1–69.1%]) goat serum samples scored positive to MAT for anti-*Toxoplasma *IgG. In particular, prevalence values in single farms in decreasing order were as follows: 42/59 (71.2% [59.6–82.7%]) in farm I, 8/13 (61.5% [35.1–88%]) in farm V, 4/7 (57.1% [20.5–93.8%]) in farm IV, 10/20 (50% [28.1–71.9%]) in farm VI, 7/15 (46.7% [21.4–71.9%]) in farm II, and 6/13 (46.1% [19-73.2%]) in farm III. Anti-*Toxoplasma *Ig titers ranged from 1 : 20 to 1 : 163840 in farm I, 1 : 20 to 1 : 80 in farm II, 1 : 40 to 1 : 1280 in farm III, 1 : 40 to 1 : 640 in farm IV, 1 : 20 to 1 : 1280 in farm V, and 1 : 20 to 1 : 160 in farm VI. Despite of these serological results, there was no clinical evidence of toxoplasmosis in the infected goats at the time of sampling.

### 3.2. Molecular Detection in Blood and Milk

Among seropositive goats (*n* = 77), the n-PCR assay gave positive results on blood samples in 10 of them. The kappa coefficient for the association between results of n-PCR on blood samples and results of n-PCR on milk samples was 1. Therefore, there was a perfect level of agreement between the two n-PCR assays in all 10 animals, indicating that 13% (5.5–20.5%) of seropositive goats and 100% of those testing positive to n-PCR on blood were excreting *T. gondii* DNA in their milk. Antibody titers in these goats were 1 : 10240 (*n* = 2), 1 : 2560 (*n* = 1), 1 : 640 (*n* = 1), 1 : 320 (*n* = 1), 1 : 160 (*n* = 1), 1 : 80 (*n* = 2), or 1 : 20 (*n* = 1). As none of the negative controls were positive, we concluded that contamination of the master mix and DNA extraction reagents had not occurred. 

### 3.3. Genotyping

Genetic characterization of DNA isolates revealed the occurrence of genotype III from 7 samples, genotype I from one sample, and atypical genotypes with hints for genotype I from 2 samples, as shown in Table 1.

## 4. Discussion

Studies on natural infections in dairy animals are important since they report what has actually occurred in farms. Our results show that exposure of dairy goats to *T. gondii* infection in the geographic area examined may be very common, since an overall prevalence of 60.6% as well as prevalence values from 46.7% to 71.2% in single farms were detected by MAT. Supposedly, goats acquired *Toxoplasma* infection on pastures. Thus, it can be hypothesized that the caprine population screened in this survey had relatively high chance to come into contact with infective *T. gondii* oocysts due to high environmental pressure. Data on the prevalence of toxoplasmosis in goat populations during 1990–1999 have been reviewed, with values reaching up to 92% in many regions of the world and ranging from 19% to 77% in Europe [9]. In Italy, a large-scale serological screening in goat farms showed that 5.6% and 12.3% of goats had IgM or Ig G antibodies specific to *T. gondii* [21]. Among abortion samples, *T. gondii* DNA was detected in 13% of fetuses and 25% of placentae from goats [22]. Thus, prevalence values found in this survey fall within those previously reported in the literature for European countries but are considerably higher than previous reports in other geographical areas of Italy. Differences in prevalence values may be dependent on several factors, including density of infected cats shedding oocysts and contaminating the environment, different susceptibility of breed to the infection, rearing conditions, and climatic situation, since oocysts are known to survive longer in moist cool conditions [23]. 

This survey documents that *T. gondii *DNA can be present in the milk of naturally infected goats, since DNA was found in 10 milk samples from 77 goats with serology positive to this parasite. PCR methods are considered to lack sensitivity due to the scanty amount of sample used for DNA extraction [24]. In addition, a positive PCR does not demonstrate the presence of viable tachyzoites or cysts containing bradyzoites, since the established reference method for detecting *Toxoplasma* from foodstuff is gavage or inoculation using either mice or cats. However, we attempted to increase sensitivity by concentration of 50 mL milk samples. Bioassay is a laborious and time-consuming technique, and it is not suitable for routine monitoring activities on large numbers of samples, also from an animal ethics point of view [25]. Moreover, the occurrence of tachyzoites was reported in the milk of several hosts, including goats [9, 11]. In particular, the finding and survival of *T. gondii* tachyzoites in goat milk were experimentally studied by Walsh and coworkers [26]. These authors observed that tachyzoites can survive in caprine milk from three to seven days at +4°C, thus providing evidence that the raw goat milk can serve as a source of toxoplasmosis. Though tachyzoites are not generally considered as an important source of oral transmission of *T. gondii *because they are rapidly killed outside the host and because they are considered sensitive to proteolytic enzymes, the possibility of infection by tachyzoites penetration of the oral-pharyngeal mucosa has been reported [13]. Tachyzoites were also shown to survive up to 2 h in pepsin solutions, and adult cats became infected when high numbers of tachyzoites were given orally [11]. Moreover, a recent work showed that DNA of viable* T. gondii* was present in 10% of goat milk samples, being able to infect both cell lines and bioassayed cats [27]. Therefore, based on the results of the present survey and on those of other studies, the possibility of *Toxoplasma* transmission through consumption of raw milk and its unpasteurized derivatives can be hypothesized and have an impact on public health [28]. 

One of the most important aims of diagnostic tests in animal production is to help to control the introduction of human pathogens into the food chain, including *T. gondii*. Therefore, the ability of molecular tests as the n-PCR assay to detect goats that are more likely to shed *T. gondii* tachyzoites into their milk is crucial. Based on the results of the present survey, goats testing positive to n-PCR on blood samples had positive results also to n-PCR on milk samples in 100% of the cases (*n* = 10). Consequently, it could be argued that a given n-PCR outcome on blood samples is conclusive as to whether or not a given seropositive goat is shedding *Toxoplasma* DNA into its milk. However, further investigations are needed to confirm the ability of blood n-PCR as indicator of the likelihood of milk shedding of *Toxoplasma* tachyzoites in dairy goats.

Results of genetic characterization showed that classical clonal type isolates (III and I) were the most commonly found in this survey. As far as we know this is the first report of genotyping of *T. gondii *isolates in goats in Italy. In Europe, the population structure of this cosmopolitan parasite is markedly clonal with a huge predominance of strains belonging to the type II lineage, which accounts for more than 95% of isolates, both in humans and animals [29–32]. Strains belonging to the type III can be observed in some cases [31], whereas the type I lineage and nonclonal (also called atypical) strains, that do not fit into the three major lineages, are exceptionally found [33, 34]. In particular, nonclonal strains of *T. gondii* have so far been reported in chickens in Poland [35], in free-ranging arctic foxes (*Vulpes lagopus*) in the Svalbard Islands [30], in oocysts from domestic cats in Germany [32], and in a captive Bennett's wallaby (*Macropus rufogriseus*) in Spain [36]. Interestingly, two isolates from Polish chickens were identical to a nonclonal sheep isolate from Uruguay [35]. Knowledge on the prevalence of atypical strains is of paramount importance. Indeed, they have also been isolated in immunocompetent human patients with severe acquired toxoplasmosis [34, 37, 38], such as the recently described “Amazonian toxoplasmosis” reported in French Guiana [39]. This is characterized by highly pathogenic and atypical strains linked to a neotropical forest-based cycle involving wild cats and their prey [39]. Moreover, there is evidence that atypical strains are more pathogenic in congenital toxoplasmosis than type II strains [34, 38, 40] and that they can lead to reinfection in pregnant women with a past immunity to type II strains [38]. In our study, the presence of two atypical strains in goats is difficult to explain and their origin remains unknown. Residence abroad or consumption of meat (especially horse meat) from countries where atypical strains are common has been suggested as possible sources of infection for clinical toxoplasmosis in humans [38, 41]. However, the two examined goats were born on the farm and were not fed with protein supplement of animal source. It has been demonstrated that sexual recombination may yield progeny with very different biological characteristics by reshuffling existing alleles between two parents [42]. Therefore, one possible explanation might be that atypical strains had emerged after a recombination event in cats sharing the same area with goats and that caprine infection occurred through the ingestion of sporulated oocysts from food or water contaminated by feces of these cats. 

## 5. Conclusion

Toxoplasmosis is a major public health issue and continues to pose a risk to public health due also to its presence in goat milk. Demands of consumers for pathogen-free products have focused the attention of government regulators and food industry on the necessity to have safe, and high-quality products. Commercial sale and/or distribution of raw milk varies among different countries. Nevertheless, unpasteurized dairy products have gained popularity by consumers who say raw milk strengthens the immune system and provides other health benefits, although it is proven that raw goat's milk is a vehicle for pathogen transmission [43]. Toxoplasmosis transmission by unpasteurized milk can represent a significant means of contamination by this agent [44, 45]. A European multicentre case-control study investigating the sources of *Toxoplasma* infection in pregnant women corroborated this evidence, reporting that in Lausanne 14% of infections were attributed to consumption of inadequately processed milk or milk products, whereas the population attributable fraction was up to 5% [33]. Our results suggest that, when n-PCR positive results on blood samples from seropositive goats are found, the risk of excretion of *Toxoplasma* tachyzoites in their milk might frequently occur. Potential transmission of *T. gondii* from the goat milk was also reported by other authors [12–14, 26, 27]. Current consumer recommendations to avoid drinking raw milk or eating fresh dairy products made from raw milk are consistent with the findings of the present study.

## Figures and Tables

**Table 1:** Multilocus genotyping of *Toxoplasma gondii* isolates from a goat population in Italy.

Goat no.	Genetic markers											
	SAG1	3′SAG2	5′SAG2	SAG2 new	SAG3	BTUB	C22-8	C29-2	GRA6	L358	PK1	Apico
1	II/III	I/III	III	III	III	III	III	III	III	NA	III	III
2	II/III	I/III	NA*	III	III	III	NA	III	NA	III	NA	III
3	II/III	I/III	III	III	III	III	III	III	NA	NA	III	NA
4	I	I/III	I/II	I	III	NA	I	NA	NA	NA	NA	I
5	II/III	III	III	III	III	NA	III	III	III	NA	III	NA
6	II/III	I/III	I/II	III	NA	III	III	III	NA	III	III	III
7	II/III	I/III	I/II	NA	III	III	NA	III	NA	III	NA	NA
8	I	I/III	III	I	III	NA	I	I	I	NA	NA	III
9	II/III	I/III	I/II	III	III	III	NA	III	NA	III	III	III
10	I	I/III	I/II	I	I	NA	I	I	I	NA	NA	I

*NA: not amplified.
